# Assessment of the efficacy and safety of anti-sclerostin antibody therapy for osteoporosis in postmenopausal women: a systematic review and meta-analysis of randomized controlled trials

**DOI:** 10.3389/fendo.2025.1732708

**Published:** 2026-01-19

**Authors:** Lianzhi Chen, Qingwen Wang, Mingxi Gu

**Affiliations:** 1 Department of Rheumatism and Immunology, Peking University Shenzhen Hospital, Shenzhen, Guangdong, China; 2 Shenzhen Key Laboratory of Inflammatory and Immunologic Diseases, Shenzhen, Guangdong, China

**Keywords:** anti-sclerostin antibodies, blosozumab, meta-analysis, osteoporosis, romosozumab

## Abstract

**Objective:**

Anti-sclerostin antibodies are novel biologics for the treatment of postmenopausal osteoporosis, while their efficacy and safety are yet to be fully understood. The aim of this systematic review and meta-analysis is to evaluate the efficacy and safety of anti-sclerostin antibodies compared to placebo, alendronate, teriparatide and denosumab in the treatment of osteoporosis.

**Methods:**

This systematic review and meta-analysis included a total of 10 randomized controlled trials (RCTs),involving 12,384 participants with postmenopausal osteoporosis, comparing anti-sclerostin antibodies with alendronate, teriparatide, denosumab, or placebo in postmenopausal women with osteoporosis. The quality of randomized controlled trials was evaluated by using the Cochrane Collaboration’s Randomized Controlled Trial Risk of Bias Assessment Tool, and meta-analysis was performed by using the RevMan software. The primary outcome was the percentage change in bone mineral density(BMD)at 6 and 12 months compared to baseline. Secondary outcomes included the incidence of adverse events and cardiovascular complications.

**Results:**

Compared with placebo, alendronate, and teriparatide, anti-sclerostin antibodies significantly increased BMD at the lumbar spine, total hip, and femoral neck at 6 and 12 months. Compared with denosumab, anti-sclerostin antibodies significantly increased lumbar spine bone mineral density at 6 months (MD = 3.68, 95% CI: 0.34-7.01, P = 0.03) and 12 months (MD = 5.20, 95% CI: 3.19–7.21, P < 0.00001). No significant differences in BMD were found at the total hip and femoral neck versus denosumab. Regarding safety, anti-sclerostin antibodies had a lower incidence of adverse events than alendronate (RR = 0.96, 95% CI: 0.93–0.99, P = 0.02) but a higher incidence than teriparatide (RR = 1.13, 95% CI: 1.01–1.25, P = 0.03). There was no significant difference in adverse events compared to placebo (RR = 0.98, 95% CI: 0.96–1, P = 0.1) or denosumab (RR = 2.64, 95% CI: 0.74–9.36, P = 0.13). Importantly, anti-sclerostin antibodies did not significantly increase the risk of cardiovascular complications compared to other treatments (RR = 1.23, 95% CI: 0.92–1.64, P = 0.17).

**Conclusion:**

Anti-sclerostin antibodies are effective at increasing BMD, with a pronounced effect on the lumbar spine, and demonstrate a controllable overall risk profile. The study results demonstrate that anti-sclerostin antibodies can be used to treat postmenopausal osteoporosis.

**Systematic Review Registration:**

https://www.crd.york.ac.uk/PROSPERO/recorddashboard, identifier CRD420251103597.

## Introduction

Osteoporosis is a systemic skeletal disease characterized by low bone mass, damage to bone microstructure, increased bone fragility, and a dramatic increase in the risk of fractures. It typically occurs in older adults, particularly postmenopausal women. With the acceleration of global aging, it has become a significant challenge in global public health (1). The key pathology of osteoporosis lies in the imbalance between bone resorption and bone formation (2). Reducing bone resorption and increasing bone formation are primary approaches for treating osteoporosis (3). Currently, most available medications are anti-resorptive agents, such as bisphosphonates, calcitonin, estrogen and estrogen analogues, and denosumab; while the only medication that promotes bone formation is the active 1–34 amino acid fragment of parathyroid hormone (e.g., teriparatide and abaloparatide) (4). However, no medication has a dual effect on bones. Therefore, developing new therapeutic drugs with potent bone-forming effects for this critical stage offers hope for overcoming existing treatment bottlenecks and achieving improved bone density and reduced fracture risk.

Research has found that sclerostin secreted by osteocytes can antagonize Wnt signaling by competitively binding to LRP5 or LRP6, thereby stabilizing β-catenin, reducing signal transduction, and decreasing osteogenic activity, which leads to osteoporosis (5). These observations promoted the development of monoclonal antibodies against sclerostin. Anti-sclerostin antibodies specifically neutralize and eliminate sclerostin, reducing its binding to LRP5/6 and lifting its inhibition of the Wnt signaling pathway, thereby strongly activating osteoblasts and significantly promoting bone formation (6).

Among numerous sclerostin inhibitors, romosozumab and blosozumab stand out as representative drugs, distinguished by their unique mechanisms of action and notable clinical efficacy, making them the most prominent focal points in osteoporosis drug development research (7). Romosozumab is a humanized anti-osteocalcin antibody that was approved in 2019 for the treatment of postmenopausal osteoporosis (brand name Evenity, Amgen and UCB). It has been approved for marketing in multiple countries (including the United States, the European Union, and Japan) for the treatment of postmenopausal women at high risk of fractures due to osteoporosis (8).In China, romosozumab has completed Phase III clinical trials for postmenopausal women with osteoporosis. In Japan, romosozumab has also been approved for treating male osteoporosis. However, the U.S. Food and Drug Administration (FDA) has suspended romosozumab use in patients with recent myocardial infarction or stroke and advises caution in patients with higher cardiovascular risk.

Its approval is based on the exceptional efficacy demonstrated in landmark Phase III clinical trials (such as the FRAME (9) and ARCH (10)studies): during the initial treatment period (first 12 months), it not only achieves unprecedented increases in bone mineral density (BMD)), but also exhibits dual effects of inhibiting bone resorption, significantly reducing the risk of vertebral and non-vertebral fractures. The therapeutic strategy of maintaining anti-resorptive effects of romosozumab beyond the “osteoanabolic window” demonstrates significant potential for sustained bone density enhancement and fracture risk reduction. As a drug targeting the same pathway, blosozumab is an IgG4 anti-sclerostin antibody (11). Although it is still in the clinical development phase and has not yet been approved by the U.S. Food and Drug Administration, the efficacy of blosozumab has been validated in three Phase 2 clinical trials, by significantly increasing the lumbar and hip bone density in postmenopausal women with osteoporosis (12).

The comparative effects of anti-sclerostin antibodies and the traditional anti-osteoporosis medications in postmenopausal women with osteoporosis have not been fully investigated. The risks of anti-sclerostin antibodies, including the serious cardiovascular adverse events, are also yet to be elucidated. Therefore, the aim of this systematic review and meta-analysis was to define the efficacy and safety of anti-sclerostin antibodies in the treatment of osteoporosis in postmenopausal women, by integrating the latest randomized controlled trials that compared the anti-sclerostin antibodies (romosozumab and blosozumab) with alendronate, teriparatide, denosumab, or placebo in postmenopausal women with osteoporosis.

## Methods

### Agreement

This systematic review and meta-analysis complied with the Preferred Reporting Items for Systematic Reviews and Meta-Analyses (PRISMA) statement guidelines (13). The design of the meta-analysis has been registered in the PROSPERO registry (CRD420251103597). This study was conducted in accordance with the Cochrane Intervention System Assessment Manual.

### Search strategy

Randomized controlled trials (RCTs) comparing anti-sclerostin antibodies with alendronate, teriparatide, denosumab, or placebo in postmenopausal women with osteoporosis were searched from PubMed, EMBASE, Web of Science, and Cochrane Central Register of Controlled Trails (CENTRAL) from their inception up to June 30, 2025 by using Medical Subject Headings terms “anti-sclerostin antibody”, “romosozumab”, “blosozumab”, “AMG 785”, “LY2541546”, and “osteoporosis”. Two researchers independently searched for and screened eligible studies, assessed the risk of bias, and extracted data from each study. The quality of randomized controlled trials was evaluated using the Cochrane Collaboration’s Randomized Controlled Trial Risk of Bias Assessment Tool, and meta-analysis was performed using review management software (RevMan, version 5.4).The primary endpoint for evaluating the efficacy of osteoporosis treatment in this study was the percentage change in bone mineral density(BMD) at 6 and 12 months compared to baseline. Secondary outcomes were the incidence of adverse events and cardiovascular complications during treatment with antiosteoporosis drugs.

### Eligibility criteria

**Inclusion Criteria:** ① Study Population: Studies involving postmenopausal women aged 50 years or older ② Intervention Measures: The experimental group used anti-sclerostin antibodies, primarily romosozumab or blosozumab, while the control group used placebo, alendronate, teriparatide, or denosumab ③ Study type: Randomized controlled trial. ④At least one of the following evaluation criteria (1): Percent change in lumbar spine BMD at 6 months (2); Percent change in total hip BMD at 6 months (3); Percent change in femoral neck BMD at 6 months (4); Percent change in lumbar spine BMD at 12 months (5); Percent change in total hip BMD at 12 months (6); Percent change in femoral neck BMD at 12 months (7); Adverse events (8); Cardiovascular Events.

**Exclusion Criteria**: ① Studies of the cohort study/retrospective study type without a control group; ② Literature lacking detailed data on the required outcome measures; ③ Literature with duplicate publications were treated as a single publication for data extraction and statistical analysis; ④ Animal experiments and finite element simulation studies; ⑤ Studies involving male subjects, young women during menstruation, or literature for which the full text was unavailable.

### Extraction of the relevant data

Two researchers independently extracted data from eligible studies. To ensure consistency in data extraction from each study, a structured table was used. Information extracted from each study included author names, publication year, study design, sample size, patient characteristics (age, gender, T-score), drug dosage, follow-up duration, and outcomes. Any discrepancies between the two researchers were resolved through consultation with a third corresponding author researcher to reach a consensus. If selected articles contained data from two or more groups, only the data to be analyzed were extracted. If the data in the included literature were incomplete, attempts were made to contact the authors to obtain raw data to include more patients in this analysis and reduce error. If the original data are not provided in the paper and the authors cannot be contacted, the data will be obtained from the original line graphs using GETDATA software. The primary outcome is the percentage change in BMD at 6 months and 12 months compared to baseline following osteoporosis treatment. The secondary outcome is the incidence of adverse events during treatment with osteoporosis medications.

### Quality assessment

Two researchers independently assessed the risk of bias in the included randomized controlled trials using the Cochrane Handbook’s risk of bias assessment tool for randomized controlled trials; The quality of randomized controlled clinical trials was assessed using the following seven criteria: random sequence generation, allocation concealment, blinding of investigators and participants, blinding of outcome assessors, incomplete outcome data, selective reporting of outcomes, and other sources of bias. The overall quality assessment for each study was categorized as “low risk of bias,” “high risk of bias,” or “unclear risk of bias.”

### Statistical analysis

Meta-analysis was performed by using literature review analysis software (RevMan, version 5.4), and the risk of publication bias was assessed by using a funnel plot of the included studies based on the outcome measurement. Continuous variables were analyzed using the inverse variance method. The mean difference (MD) was used to assess the effect size of the samples, with a 95% confidence interval (CI). For dichotomous variables, the results were expressed as relative risk (RR) as the effect measure, with the confidence interval also set at 95%. The I² index was used to assess heterogeneity. An I² value below 25% was considered homogeneous, while values between 25% and 75% and 75% or above were classified as moderate and high heterogeneity, respectively. A fixed-effects model was used for homogeneous studies, while a random-effects model was employed when moderate or high heterogeneity was present. Sensitivity analyses or subgroup analyses may be conducted to investigate the sources of heterogeneity, thereby obtaining reliable conclusions and assessing the stability of the results. The significance level was set at α = 0.05.

## RESULTS

### Search results and study characteristics

**Figure 1:**
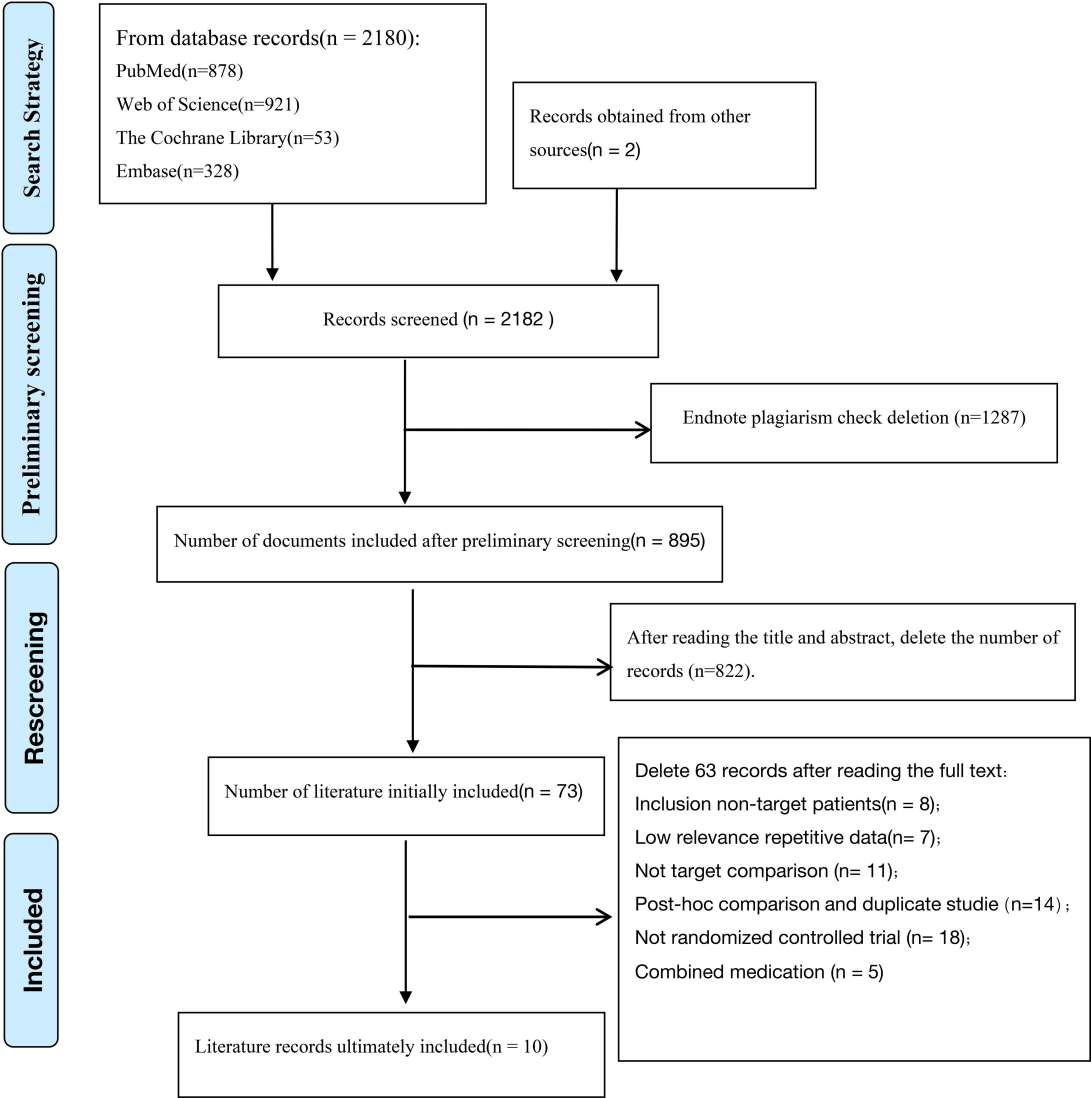
Flow chart summarizing the selection process.

A total of 2,182 articles were retrieved from the PubMed, Embase, Web of Science, and Cochrane Library databases. After importing the literature into EndNote X9 software for duplicate removal, 1,287 articles were excluded; an additional 822 articles were excluded based on their titles and abstracts, leaving 73 articles initially included for full-text assessment. After carefully reviewing the full texts, 63 articles were excluded for failing to meet the selection criteria. Finally, ten RCTs involving 12,384 patients were included in the systematic review and meta-analysis (14–23). **Figure 1** summarizes the literature search and screening process. The primary characteristics of the included study were listed in **Table 1**.

**Table 1:** Primary characteristics of the included studies.

Author	Year	Location	Study design	Anti-sclerostin antibodies	Treatment		Sample size		Mean age (years)		T-score	Duratio (months)	Outcome
					Anti-sclerostin antibodies	Control	Anti-sclerostin antibodies	Control	Anti-sclerostin antibodies	Control			
Baek et al.	2021	Korean	Phase III RCT	Romosozumab	Romosozumab 210 mg sc monthly	Placebo	34	33	66.7	68.4	-4≤T≤-2.5	6	[1, 3, 5, 7, 8]
Cosman et al.	2016	USA	Phase III RCT	Romosozumab	Romosozumab 210 mg sc monthly	Placebo	3589	3591	70.9	70.8	-3.5≤T≤-2.5	12	[1, 2, 3, 4, 5, 6, 7, 8]
Langdahl et al.	2017	America, Europe	Phase III RCT	Romosozumab	Romosozumab 210 mg sc monthly	Teriparatide 20ug sc daily	218	218	71.8	71.2	-3.5≤T≤-2.5	12	[1, 2, 3, 4, 5, 6, 7]
Ishibash et al.	2017	Japan	Phase II RCT	Romosozumab	Romosozumab 210 mg sc monthly	Placebo	63	63	68.3	67.8	-4≤T≤-2.5	12	[1, 2, 3, 4, 5, 6, 7]
Saag et al.	2017	Worldwide	Phase III RCT	Romosozumab	Romosozumab 210 mg sc monthly	Alendronate 70mg po weekly	2046	2047	74.4	74.2	T<-2	12	[1, 2, 3, 4, 5, 6, 7]
Mochizuki et al.	2021	Japan	Phase II RCT	Romosozumab	Romosozumab 210 mg sc monthly	Denosumab 60mg sc, every 6 months	25	25	74	73	T≤-2.5	6	[1, 3, 5]
Mochizuki et al.	2022	Japan	Phase II RCT	Romosozumab	Romosozumab 210 mg sc monthly	Denosumab 60mg sc, every 6 months	26	25	74.8	72	T≤-2.5	12	[2, 4, 6, 7]
Recker et al.	2015	Worldwide	Phase II RCT	Blosozumab	Blosozumab 270 mg sc every 2 weeks	Placebo	30	29	66.1	66	-3.5≤T≤-2.5	12	[1, 2, 3, 4, 7, 8]
McClung et al.	2014	Worldwide	Phase II RCT	Romosozumab	Romosozumab 210 mg sc monthly	Placebo	52	52	66.3	67	-3.5≤T≤-2	12	[1, 2, 3, 4, 5, 6, 7]
McClung et al.	2014	Worldwide	Phase II RCT	Romosozumab	Romosozumab 210 mg sc monthly	Alendronate 70mg po weekly	52	51	66.3	67.1	-3.5≤T≤-2	12	[1, 2, 3, 4, 5, 6, 7]
McClung et al.	2014	Worldwide	Phase II RCT	Romosozumab	Romosozumab 210 mg sc monthly	Teriparatide 20ug sc daily	52	55	66.3	66.8	-3.5≤T≤-2	12	[1, 2, 3, 4, 5, 6, 7]
Sobue et al.	2025	Japan	Phase III RCT	Romosozumab	Romosozumab 210 mg sc monthly	Denosumab 60mg sc, every 6 months	56	56	81.3	80.4	T<-2.5	12	[1, 2, 3, 4, 5, 6, 7, 8]

[1] Percent change in lumbar spine BMD at 6 months.

[2] Percent change in lumbar spine BMD at 12 months.

[3] Percent change in total hip BMD at 6 months.

[4] Percent change in total hip BMD at 12 months.

[5] Percent change in femoral neck BMD at 6 months.

[6] Percent change in femoral neck BMD at 12 months.

[7] Adverse events.

[8] Cardiovascular Events.

### Risk of bias in included studies

**Figure 2:**
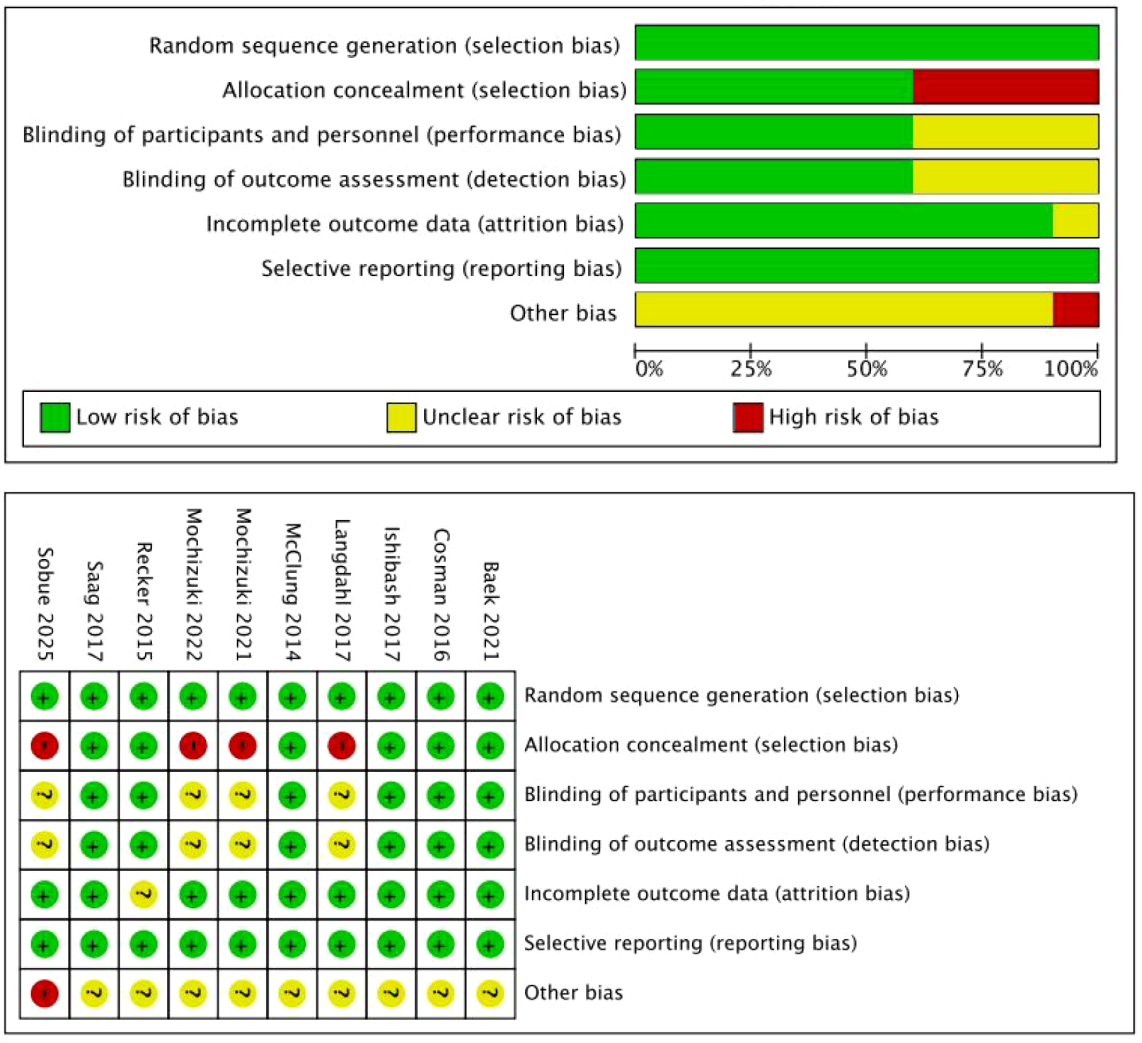
Summary of the risk of bias of the RCTs included in the meta-analysis.

A literature quality assessment of randomized controlled trials was conducted by using the risk and bias tables in RevMan 5.4 software. The risk of bias in the included RCTs is presented in **Figure 2**. All ten studies described how the random sequences were generated. Therefore, their studies were assessed as low risk. Six randomized trials (14–16, 18, 19, 22)used the same drug and the same packaging, which did not affect the trials, so we classified them as low risk. Four studies (17, 20, 21, 23) were open-label studies without allocation concealment and were at high risk of selection bias, posing an unclear risk of selection bias. Six studies (14–16, 18, 19, 22) reported adequate blinding of investigators, participants, and outcome assessors, while three studies (17, 20, 21) did not describe blinding of outcome assessors, and one study (23) did not use blinding. Nine of the included randomized (14–21, 23) controlled trials fully reported data on the primary outcome. In Recker et al.’s study (22), bias due to missing outcome data was also a concern, with some data obtained from original line graphs using GETDATA software. In all included studies, the risk of bias from the randomization process, outcome measurement bias, and reporting outcome selection bias was assessed as low. All randomized trials included in the analysis reported results for the pre-specified measures, with no high risk of bias. One study (23) reported a high risk of bias from other sources, as the principal investigators in Sobue et al.’s team changed during the trial period due to relocation or retirement.

### Percentage change from baseline in lumbar spine BMD at 6 months

**Figure 3:**
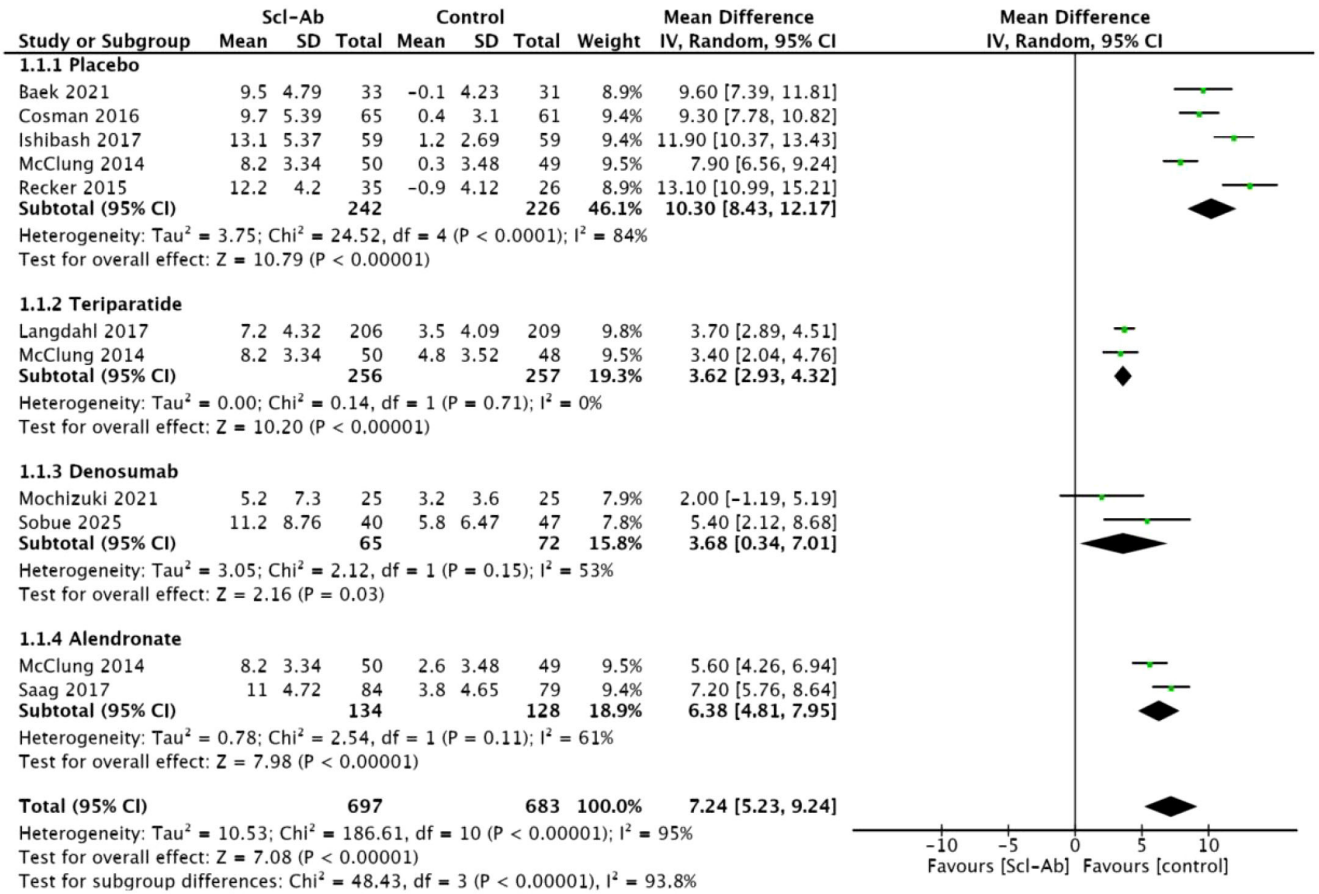
Forest plot of percentage change from baseline in lumbar spine at 6 months.

Nine studies (14–19, 21–23) reported the percentage change in lumbar spine BMD at 6 months compared to baseline. This meta-analysis showed a statistically significant difference between anti-sclerostin antibodies and other subgroups (MD = 7.24, 95% CI: 5.23-9.24, P<0.00001, **Figure 3**). Anti-sclerostin antibodies significantly increased lumbar spine bone mineral density at 6 months compared with placebo (MD = 10.3, 95% CI: 8.43–12.17, P < 0.00001), alendronate (MD = 6.38, 95% CI: 4.81–7.95, P < 0.00001), teriparatide (3.62, 95% CI: 2.93–4.32, P < 0.00001), and denosumab (MD = 3.68, 95% CI: 0.34–7.01, P = 0.03). However, there was high heterogeneity among the studies (I² = 95%). In subgroup analyses, the placebo subgroup showed high heterogeneity (I² = 84%), the alendronate subgroup and denosumab subgroup showed moderate heterogeneity (I² = 61%, I² = 53%), and the teriparatide subgroup showed homogeneity (I² = 0%).

### Percentage change from baseline in lumbar spine BMD at 12 months

**Figure 4:**
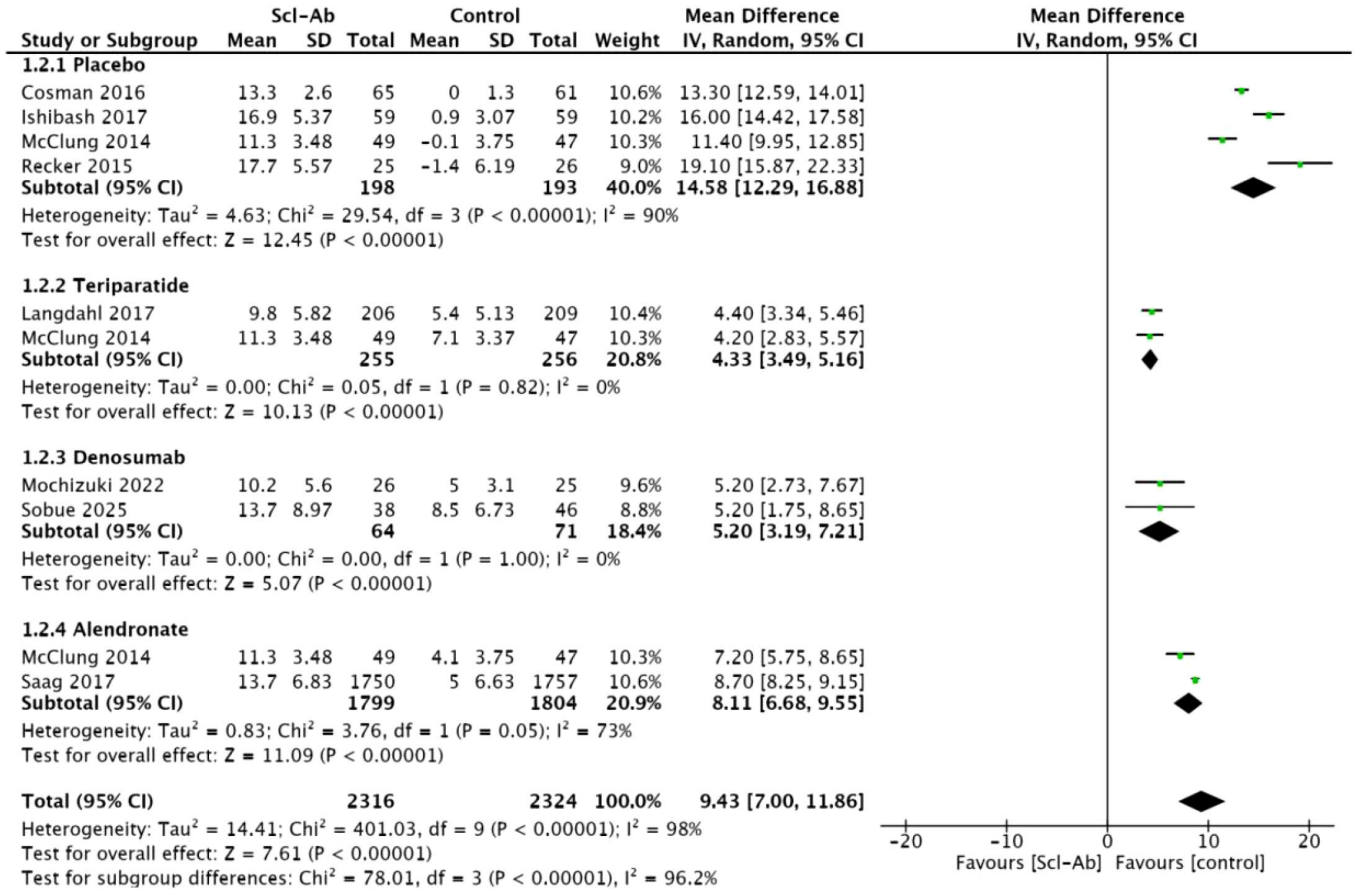
Forest plot of percentage change from baseline in lumbar spine at 12 months.

Eight studies (15–20, 22, 23) reported the percentage change in lumbar spine BMD at 12 months compared to baseline. This meta-analysis showed a statistically significant difference between anti-sclerostin antibodies and other subgroups (MD = 9.43, 95% CI: 7.00–11.86, P<0.00001, **Figure 4**). Anti-sclerostin antibodies significantly increased lumbar spine BMD at 12 months compared with placebo (MD = 14.58, 95% CI: 12.29–16.88, P < 0.00001), alendronate (MD = 8.11, 95% CI: 6.68–9.55, P < 0.00001), teriparatide (MD = 4.33, 95% CI: 3.49–5.16, P < 0.00001), and denosumab (MD = 5.20, 95% CI: 3.19–7.21, P < 0.00001). There was high heterogeneity among studies (I² = 98%). In subgroup analyses, the placebo subgroup showed high heterogeneity (I² = 90%), the alendronate subgroup showed moderate heterogeneity (I² = 73%), and the teriparatide and denosumab subgroups showed homogeneity (I² = 0%).

### Percentage change from baseline in total hip BMD At 6 months

**Figure 5:**
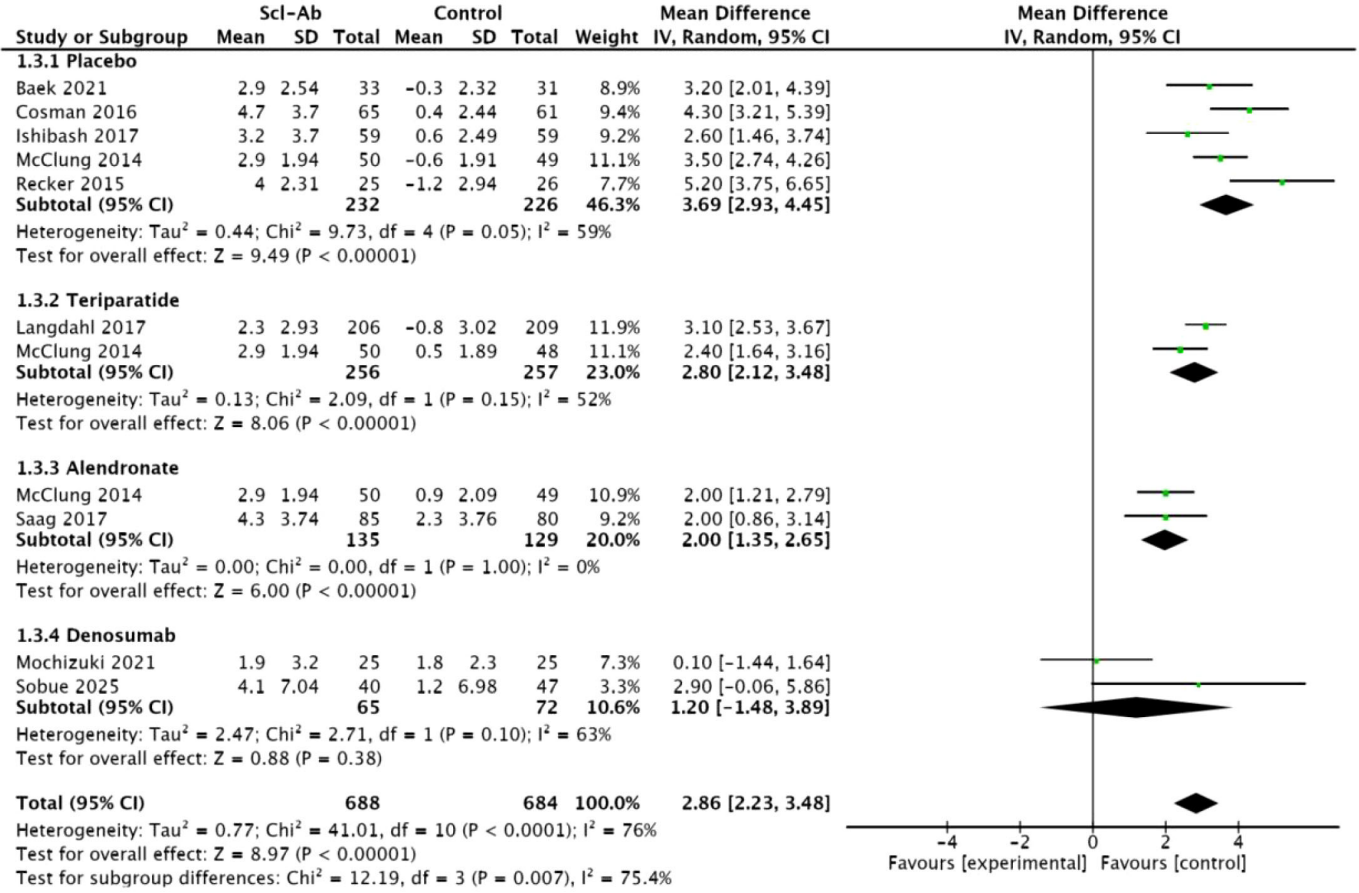
Forest plot of percentage change from baseline in total hip at 6 months.

Nine studies (14–19, 21–23) reported the percentage change in total hip BMD from baseline at 6 months. This meta-analysis showed a significant difference between anti-sclerostin antibodies and other subgroups (MD = 2.86, 95% CI: 2.23–3.48, P < 0.00001, **Figure 5**). Compared with placebo, alendronate, and teriparatide, anti-sclerostin antibodies significantly increased total hip BMD at 6 months. Placebo (MD = 3.69, 95% CI: 2.93–4.45, P < 0.00001), alendronate (MD = 2, 95% CI: 1.35–2.65, P < 0.00001), and teriparatide (2.8, 95% CI: 2.12–3.48, P < 0.00001). However, compared with denosumab, there was no significant difference in BMD at the total hip at 12 months between the two groups (MD = 1.20, 95% CI: -1.48–3.89, P = 0.38). Additionally, there was high heterogeneity among the studies (I² = 76%). In subgroup analyses, moderate heterogeneity was observed in the placebo subgroup, teriparatide subgroup, and denosumab subgroup (I² = 59%, I² = 52%, I² = 63%), while homogeneity was observed in the alendronate subgroup (I² = 0%).

### Percentage change from baseline in total hip BMD At 12 months

**Figure 6:**
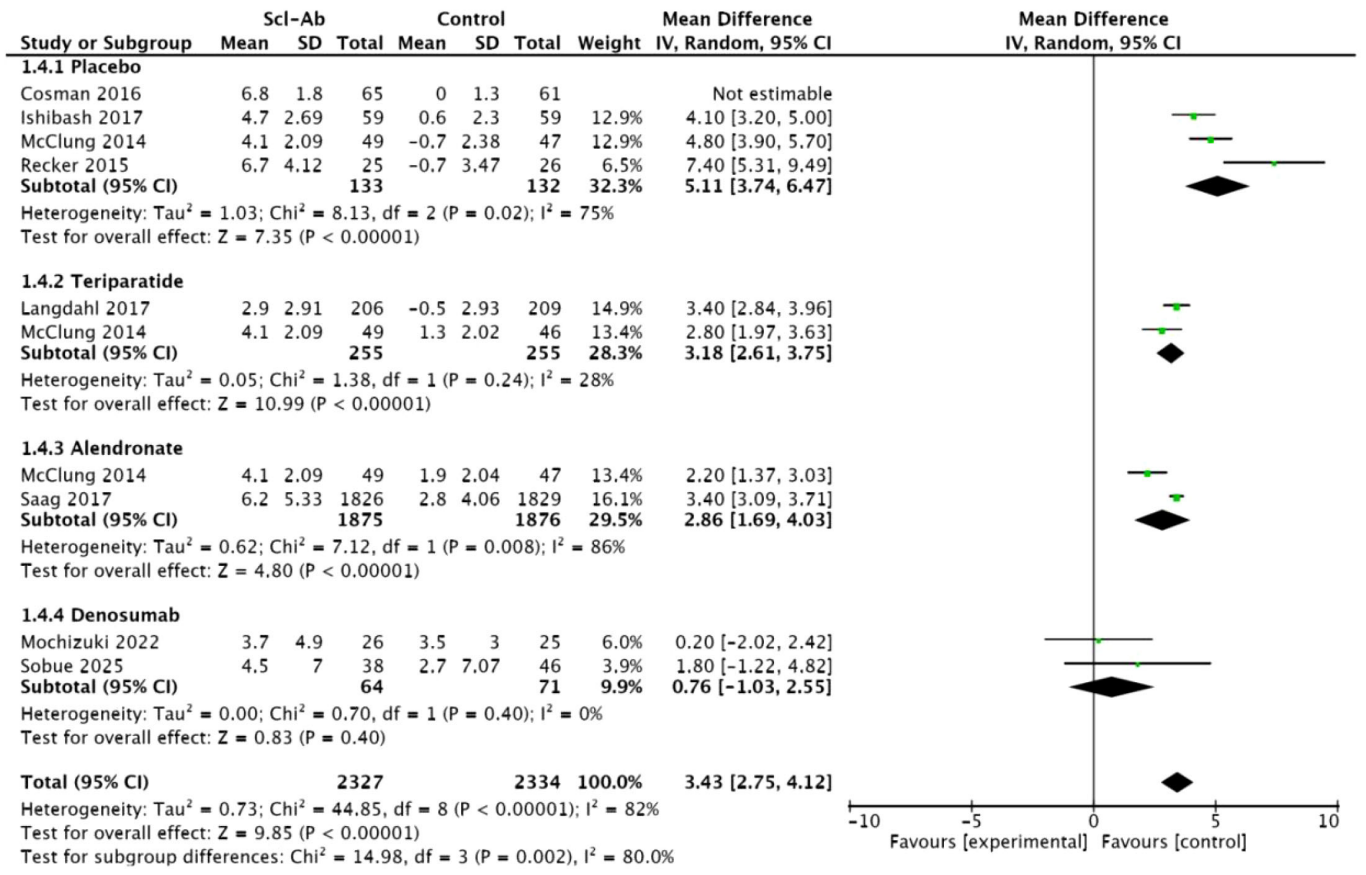
Forest plot of percentage change from baseline in total hip at 12 months.

Eight studies (15–20, 22, 23) reported the percentage change in total hip BMD from baseline at 12 months. This meta-analysis showed a significant difference between anti-sclerostin antibodies and other treatment methods (MD = 3.43, 95% CI: 2.75–4.12, P < 0.00001, **Figure 6**). Compared with placebo, alendronate, and teriparatide, anti-sclerostin antibodies significantly increased total hip BMD at 12 months, with placebo (MD = 5.11, 95% CI: 3.74–6.47, P < 0.00001), alendronate (MD = 2.86, 95% CI: 1.69–4.03, P < 0.00001), and teriparatide (3.18, 95% CI: 2.61–3.75, P < 0.00001). However, compared with denosumab, there was no significant difference in BMD at the total hip at 12 months (MD = 0.76, 95% CI: -1.03 to 2.55, P = 0.4). Additionally, there was high heterogeneity among the studies (I² = 82%). In subgroup analyses, studies in the placebo subgroup and the alendronate subgroup showed high heterogeneity (I² = 75%, I² = 86%), studies in the teriparatide subgroup showed moderate heterogeneity (I² = 28%), and studies in the denosumab subgroup showed homogeneity (I² = 0%).

### Percentage change from baseline in femoral neck BMD At 6 months

**Figure 7:**
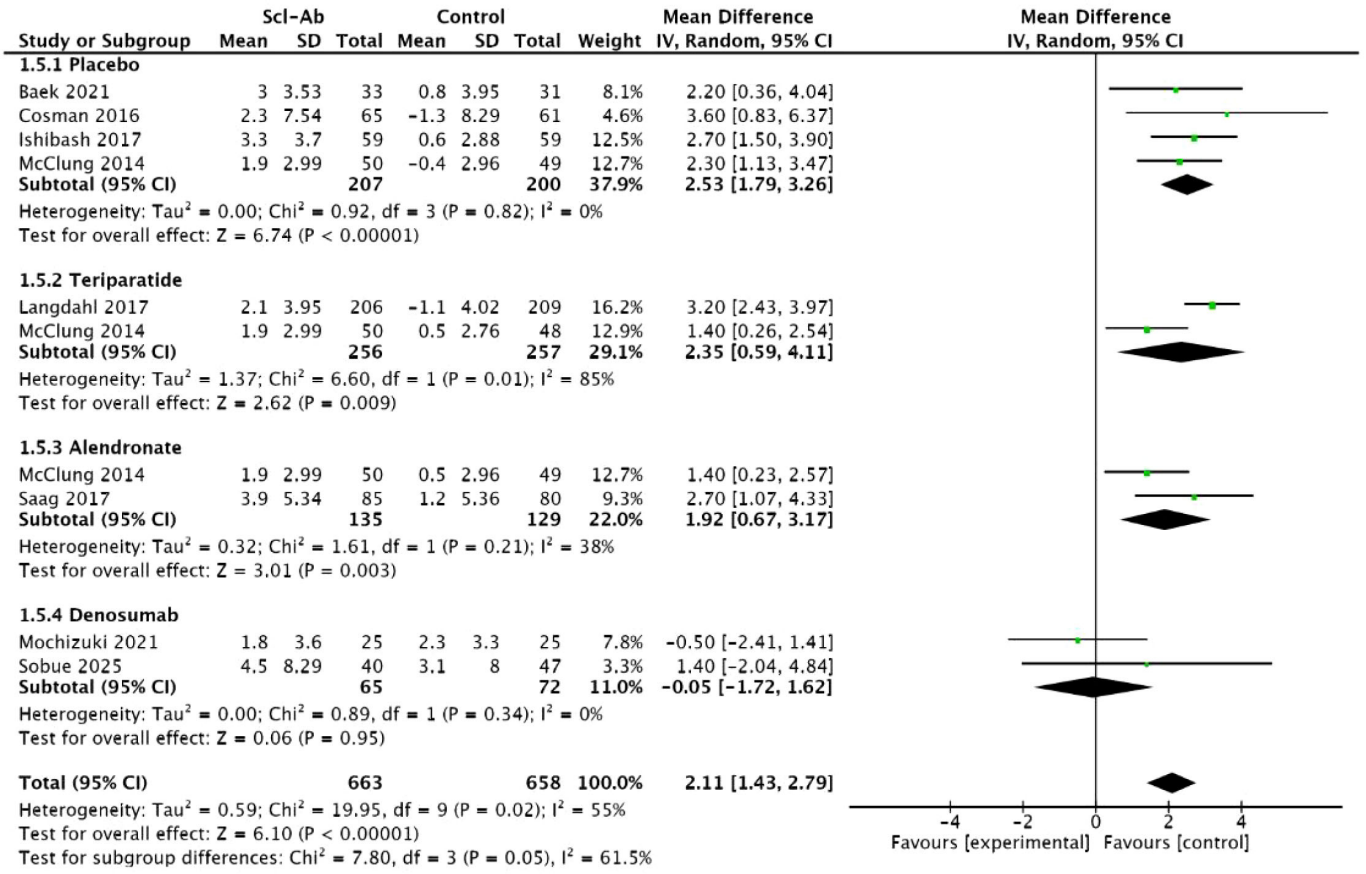
Forest plot of percentage change from baseline in femoral neck at 6 months.

Eight studies (14–19, 21, 23) reported the percentage change in femoral neck BMD from baseline at 6 months. The results of this meta-analysis showed a significant difference between anti-sclerostin antibodies and other treatment methods (MD = 2.11, 95% CI: 1.43–2.79, P < 0.00001, **Figure 7**). Compared with placebo, alendronate, and teriparatide, anti-sclerostin antibodies significantly increased femoral neck BMD at 6 months. The placebo subgroup (MD = 2.53, 95% CI: 1.79–3.26, P < 0.00001), the alendronate subgroup (MD = 1.92, 95% CI: 0.67–3.17, P = 0.003), and the teriparatide subgroup (MD = 2.35, 95% CI: 0.59–4.11, P = 0.009). Compared with denosumab, there was no significant difference in femoral neck BMD at 6 months (MD = -0.05, 95% CI: -1.72 to 1.62, P = 0.95). Moderate heterogeneity was observed between studies (I² = 55%). In subgroup analyses, the placebo subgroup and the denosumab subgroup showed homogeneity (I² = 0%), the alendronate subgroup studies showed moderate heterogeneity (I² = 38%), and the teriparatide subgroup studies showed high heterogeneity (I² = 85%).

### Percentage change from baseline in femoral neck BMD At 12 months

**Figure 8:**
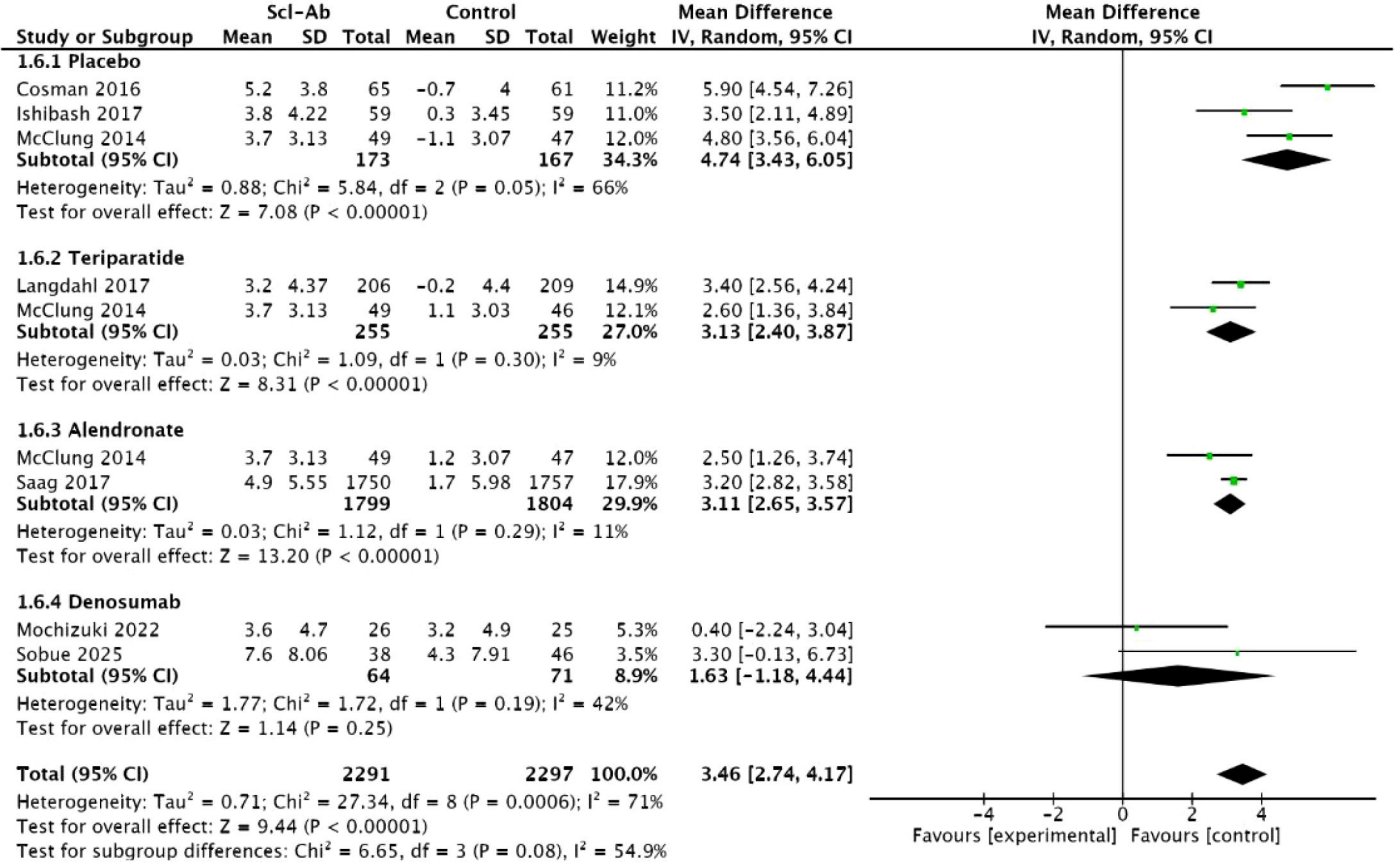
Forest plot of percentage change from baseline in femoral neck at 12 months.

Seven studies (15–20, 23) reported the percentage change in femoral neck BMD from baseline at 12 months. This meta-analysis showed a significant difference between anti-sclerostin antibodies and other treatment methods (MD = 3.46, 95% CI: 2.74–4.17, P < 0.00001, **Figure 8**). Compared with placebo, alendronate, and teriparatide, anti-sclerostin antibodies significantly increased BMD at the femoral neck at 12 months, with placebo (MD = 4.74, 95% CI: 3.43–6.05, P < 0.00001), alendronate (MD = 3.11, 95% CI: 2.65–3.57, P < 0.00001), and teriparatide (3.13, 95% CI: 2.4–3.87, P < 0.00001). Compared with denosumab, there was no significant difference in femoral neck BMD at 12 months (MD = 1.63, 95% CI: -1.18 to 4.44, P = 0.25). Subgroup analysis revealed moderate heterogeneity among studies (I² = 71%). Moderate heterogeneity was observed between studies in the placebo subgroup and the denosumab subgroup (I² = 66%, I² = 42%), while studies in the alendronate subgroup and the teriparatide subgroup showed homogeneity (I² = 11%, I² = 9%).

### Any adverse events

**Figure 9:**
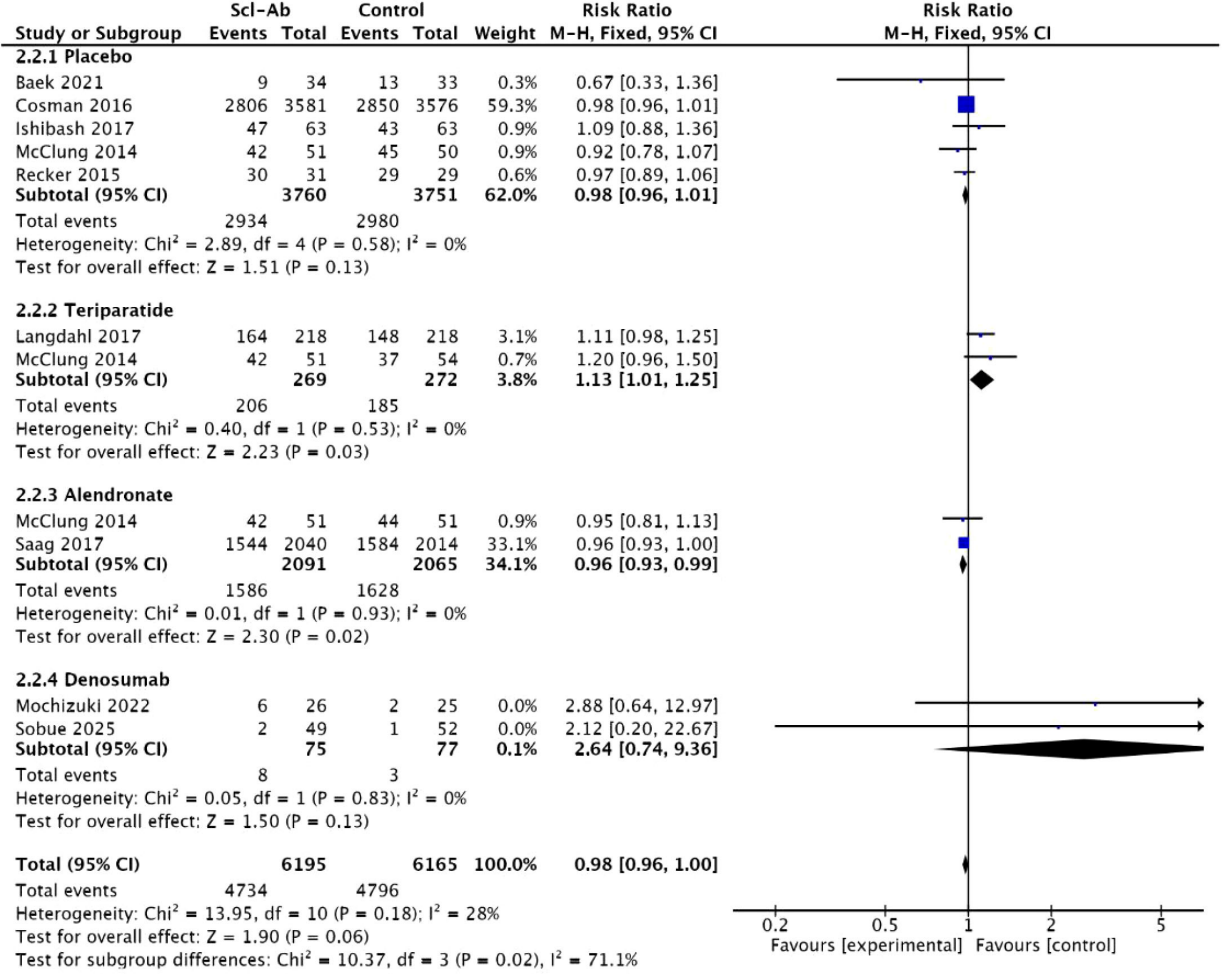
Forest plot of “any adverse events”.

Nine of the included studies (14–20, 22, 23) reported the outcome of “any adverse events,” while one study (23)only recorded serious adverse events. The meta-analysis indicated that there was no significant difference in the incidence of adverse reactions between anti-sclerostin antibodies and other studies (RR = 0.98, 95% CI: 0.96–1, P = 0.06, **Figure 9**). The anti-sclerostin antibodies subgroup showed low heterogeneity compared with the placebo, alendronate, teriparatide, and denosumab subgroups (all I²=0%), therefore a fixed-effect model was used to pool the data. Subgroup analysis results showed that compared with the alendronate subgroup, anti-sclerostin antibodies had a lower incidence of adverse events (RR = 0.96, 95% CI: 0.93–0.99, P = 0.02). However, compared with teriparatide subgroup, the anti-sclerostin antibody group had a higher incidence of adverse events (RR = 1.13, 95% CI: 1.01–1.25, P = 0.03).There were no significant statistical differences in the incidence of adverse events between anti-sclerostin antibodies and placebo or denosumab (RR = 0.98, 95% CI: 0.96–1.01, P = 0.13; RR = 2.64, 95% CI: 0.74–9.36, P = 0.13).

### Cardiovascular events

**Figure 10:**
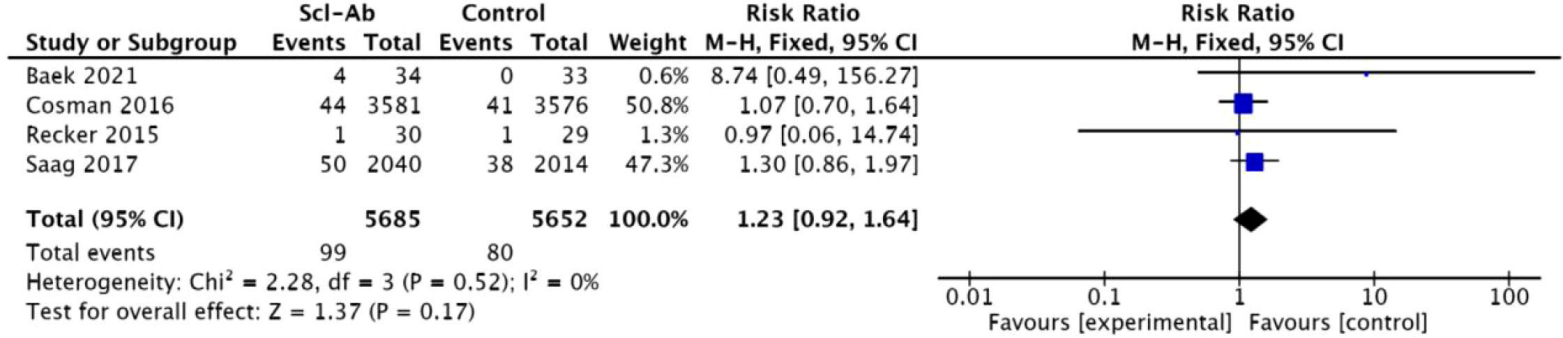
Forest plot of cardiovascular events.

Four studies (14, 16, 22, 23) documented the occurrence of “cardiovascular events” as a complication. There was homogeneity between anti-sclerostin antibodies and other osteoporosis treatments (I² = 0%), so a fixed-effect model was used to pool the data. The meta-analysis indicated that, compared with other studies, anti-sclerostin monoclonal antibodies did not significantly increase the risk of cardiovascular complications (RR = 1.23, 95% CI: 0.92–1.64, P = 0.17, **Figure 10**).

### Publication bias and sensitivity analysis

**Figure 11:**
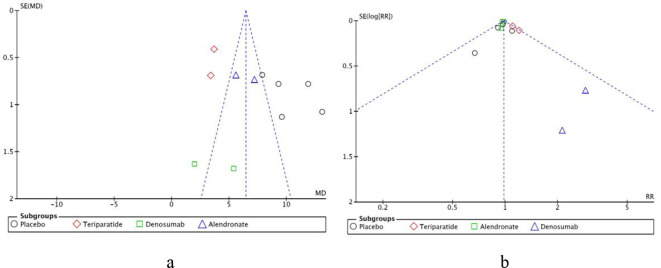
Funnel plot of publication bias. **(a)** Publication bias for primary outcome measures; **(b)** Publication bias for secondary outcome measures.

To assess publication bias for the primary outcome measures, a funnel plot was used to analyze the percentage change in lumbar BMD compared to baseline after 6 months of anti-osteoporosis treatment. While studies should be evenly distributed on either side of the vertical line, several studies fell outside the two diagonal lines, suggesting that the literature included in the assessment of the efficacy of anti-sclerostin antibodies may be subject to publication bias (**Figure 11a**). To assess publication bias for the secondary outcome measures, a funnel plot was used to analyze the incidence of adverse events during treatment with anti-osteoporosis drugs. The funnel plot exhibited a symmetrical inverted funnel shape, with most studies symmetrically distributed around the central vertical line, and all points falling within the 95% confidence interval, indicating that publication bias in the literature included for assessing the safety of anti-sclerostin antibodies was relatively low (**Figure 11b**). Overall, since only 10 studies were included in this meta-analysis, the symmetry of the funnel plot may not be accurate, making it difficult to reliably assess publication bias. Therefore, we conducted a sensitivity analysis by excluding each study individually to assess the heterogeneity and robustness of the pooled results. The results of the sensitivity analysis indicated that the results of this meta-analysis were stable.

## Discussion

Postmenopausal women experience a sharp decline in estrogen levels, leading to bone resorption significantly exceeding bone formation, making them a high-risk group for osteoporosis. Currently, there are two primary approaches to treating primary osteoporosis: one is to reduce bone resorption, and the other is to increase bone formation. The most frequently prescribed medications for treating bone loss are Bisphosphonates (BPs) and the receptor activator for the nuclear factor κB-ligand (RANKL) inhibitor denosumab (DEN).The RANK system, comprising its ligand (RANKL) and osteoprotegerin (OPG), is essential for bone remodeling. RANKL binds to RANK, triggering osteoclast precursor cell differentiation and osteoclast activity. OPG counterbalances the effects of RANKL (24). Imbalances in RANKL/OPG expression can lead to skeletal disorders such as osteoporosis. Current anti-resorptive therapies used to prevent or treat metabolic bone diseases exert their effects by modulating RANKL/OPG expression. On the other hand, Traditional anabolic agents primarily include teriparatide and abaloparatide, which regulate metabolism and promote bone formation by selectively activating the parathyroid hormone type 1 receptor signaling pathway. However, due to slow onset of action, inconvenient administration, or potential side effects, teriparatide and abaloparatide are prescribed less frequently. Sclerostin monoclonal antibodies (such as romosozumab and blosozumab) represent novel biologics targeting bone metabolic pathways. By inhibiting sclerostin activity, they promote bone formation and suppress bone resorption, offering an innovative therapeutic strategy for postmenopausal osteoporosis (25, 26). This systematic review and meta-analysis, synthesizing data from 10 randomized controlled trials (RCTs) involving 12,384 patients, provides crucial.

Sclerostin is a glycoprotein specifically secreted by osteocytes and serves as a critical negative regulator of the Wnt/β-catenin signaling pathway (26). By inhibiting this pathway, sclerostin reduces osteoblast differentiation and activity, thereby suppressing bone formation (27). Anti-sclerostin antibodies achieves a “dual effect” by specifically binding to and neutralizing circulating sclerostin, reducing its suppression effect of bone formation. It strongly promotes bone formation by stimulating the proliferation and differentiation of osteoblast precursor cells, increasing the synthesis and mineralization of bone matrix, On the other hand, it also inhibits bone resorption to a certain extent (28). This meta-analysis revealed that compared to conditional anti-resorptive agents (e.g., alendronate, denosumab) or anabolic agents (e.g., teriparatide), anti-sclerostin antibodies employ a dual-pronged strategy. Significant bone density increases were observed as early as 6 months, enabling faster bone mass enhancement. hip, and femoral neck. Theoretically, this could confer enhanced fracture resistance, with anti-resorptive effects persisting through 12 months. This rapid osteoanabolic action of romosozumab is crucial for patients who have recently suffered brittle fractures, are at extremely high risk of fracture, or require urgent bone mass enhancement (29). The ‘osteoanabolic window’ compensates for the relatively slow onset of action seen with traditional anti-resorptive drugs (30).

Denosumab is a human monoclonal immunoglobulin G2 antibody with high specificity and strong affinity for human RANKL. This antibody effectively blocks the binding of RANK to RANKL, thereby reducing bone turnover and increasing bone mass. Denosumab is currently recognized as one of the most potent anti-resorptive agents (31). This analysis found that anti-sclerostin antibodies was significantly superior to denosumab in improving lumbar spine BMD (MD = 5.20 at 12 months, P < 0.00001). This finding is of great clinical significance as the lumbar spine has metabolically active trabecular bone, which usually response to anabolic agents sensitively and rapidly (32). The efficacy of anti-sclerostin antibodies in the lumbar spine highlights its unique value as a potent osteogenic metabolic promoter, indicating greater potential in reducing the risk of vertebral fractures (33). However, no significant differences were observed between anti-sclerostin antibodies and denosumab in BMD at the total hip and femoral neck at the at 6 and 12 months. These results suggested that the advantages of anti-sclerostin antibodies may be site-specific, particularly in the lumbar spine. Compared to lumbar spine, the hip has a higher cortical bone mass, which shows lower bone turnover rates. This may explain the observation that lumbar spine showed better response to anti-sclerostin antibodies compared to hip fracture (34). This finding guides clinicians in making individualized treatment decisions by considering the patient’s primary fracture risk site. For patients with vertebral fractures as the primary risk, anti-sclerostin antibodies may be a more optimal choice.

In this study, we also conducted a multidimensional assessment to investigate the safety of anti-sclerostin antibodies. First, regarding treatment-emergent adverse events (TEAEs), anti-sclerostin antibodies showed no statistically significant difference in the overall incidence of adverse events (AEs) compared to placebo and denosumab. This preliminary finding suggested that anti-sclerostin antibodies had good overall tolerability, comparable to placebo and the traditional agents, laying the foundation for its clinical application. More favorably, compared with oral alendronate, the overall AE risk in the anti-sclerostin antibody group was even slightly reduced with statistical significance (RR = 0.96), potentially due to the avoidance of alendronate’s common upper gastrointestinal adverse reactions, representing an important advantage for patients with gastrointestinal sensitivity (35). However, compared with teriparatide, the risk of AEs in the anti-sclerostin antibody group was significantly higher (RR = 1.13). Teriparatide and romozumab are both anabolic bone-forming agents administered via subcutaneous injection, exhibiting similar adverse reactions primarily including dizziness, headache, leg cramps, nausea, and injection site reactions. Although teriparatide demonstrated an increased risk of osteosarcoma in animal studies, resulting in a black box warning for osteosarcoma, this risk has not been confirmed in current human clinical trials (36). Key clinical trials for romozumab (FRAME, ARCH) observed an increased incidence of hypocalcemia, representing a clear risk requiring active management. Romozumab exhibits a higher overall adverse event rate than teriparatide, primarily attributable to its distinct hypocalcemia risk (36).

Romosozumab has been shown in pivotal clinical trials (such as ARCH and FRAME) and post-marketing surveillance to potentially increase the risk of major adverse cardiovascular events (MACE, including myocardial infarction, stroke, and cardiovascular death), particularly in patients with pre-existing cardiovascular disease or high-risk factors (37). This may be related to the fact that sclerostin is also expressed in vascular walls (such as vascular smooth muscle cells and endothelial cells) and may be involved in the regulation of vascular calcification (38). Theoretically, inhibiting sclerostin may have potential effects on the cardiovascular system (39). The US FDA approved romosozumab with a black box warning, contraindicating its use in patients who have experienced a myocardial infarction or stroke within the past year (40). Brozomab is currently in the developmental stage, and its cardiovascular risks have not been fully elucidated. This is one of the primary safety concerns regarding the clinical application of anti-sclerostin antibodies (particularly romosozumab) at present. However, the results of a specialized analysis of cardiovascular adverse events (CVAE) in this meta-analysis showed that anti-sclerostin antibodies did not significantly increase the risk of CVAE compared to denosumab, teriparatide, and denosumab, teriparatide, and alendronate (RR = 1.23, 95% CI: 0.92–1.64, P = 0.17). This finding aligns with recent large real-world data from Stokar J and Hartz MC et al.: romosozumab not only failed to increase CVAE risk but also demonstrated a lower incidence of cardiovascular adverse events (41, 42).

There are, however, limitations in this study. Firstly, the primary endpoint of this meta-analysis was BMD, not fracture rate. BMD is a strong predictor of fracture risk and a commonly used surrogate endpoint, but reducing fracture rate is the ultimate goal of OP treatment. The follow-up period of the included studies was relatively short (≤12 months), which may not be sufficient to fully assess the long-term preventive effects of anti-sclerostin antibodies on vertebral and non-vertebral fractures (42). Secondly, potential issues with data pooling: combining the analysis of romosozumab and blosozumab may obscure potential subtle differences between the two drugs. The pooled AEs and CVAEs data cannot distinguish the severity or specific type of events, such as whether cardiovascular events are myocardial infarctions or arrhythmias. This is critical for risk assessment. Thirdly, the included studies may have differences in specific drugs, doses, treatment durations, control drug doses, and patient baseline characteristics (e.g., fracture risk stratification, bone density levels), leading to high heterogeneity. Additionally, the findings of this meta-analysis suggest that anti-sclerostin antibodies may be used in postmenopausal women with osteoporosis, while also revealing complexities regarding their safety profile. However, it is important to note that their use as a preventive agent remains far from being recommended or widely adopted. Future research urgently requires high-quality studies and large-scale real-world investigations focusing on long-term fracture outcomes, in-depth cardiovascular safety assessments, and optimized treatment strategies (such as sequential therapy). This will comprehensively establish the role of anti-sclerostin antibodies within the osteoporosis treatment landscape and guide their safe and effective application across broader, more complex clinical scenarios.

Anti-sclerostin antibody (romosozumab, blosozumab) is a novel biologic agent for the treatment of postmenopausal osteoporosis, demonstrating comprehensive and rapid advantages in improving bone mineral density (BMD), particularly in enhancing lumbar spine bone mass, thereby providing a powerful means of bone improvement for postmenopausal osteoporosis (OP) patients. The overall risk of adverse drug reactions is safe and controllable, especially for patients with high fracture risk, who require rapid bone mass improvement, and have no cardiovascular contraindications. Future studies should include large-scale, long-term real-world research with fracture as the primary endpoint to assess the long-term efficacy and safety of anti-sclerostin antibodies in broader and more complex real-world clinical settings.

## Data Availability

The original contributions presented in the study are included in the article/supplementary material. Further inquiries can be directed to the corresponding author/s.
